# Uncovering non-autoimmune hypothyroidism: A case report of rhabdomyolysis and myocarditis mimicking acute coronary syndrome

**DOI:** 10.1097/MD.0000000000042047

**Published:** 2025-04-04

**Authors:** Hyejin Jeon, Seunghye Lee, Sehyun Jung, Hani Jang, Se-Ho Chang, Hyun-Jung Kim

**Affiliations:** a Department of Internal Medicine, Gyeongsang National University Hospital, Jinju, South Korea; b Institute of Medical Sciences, Gyeongsang National University, Jinju, South Korea; c Department of Internal Medicine, College of Medicine, Gyeongsang National University, Jinju, South Korea.

**Keywords:** acute kidney injury, hypothyroidism, myocarditis, rhabdomyolysis

## Abstract

**Rationale:**

Non-autoimmune hypothyroidism is a rare but significant cause of rhabdomyolysis and myocarditis, which can mimic acute coronary syndrome. Early recognition and treatment of hypothyroidism are crucial, especially in patients with chronic kidney disease (CKD), where delayed diagnosis may lead to life-threatening complications such as acute kidney injury.

**Patient concerns:**

A 71-year-old male with diabetes, CKD stage G3aA3, and a history of chronic myelogenous leukemia presented with myalgia, anorexia, and anuria. He also reported intermittent chest pain for 1 month, progressing to severe symptoms including decreased urine output and anuria.

**Diagnoses:**

Laboratory findings revealed acute kidney injury (serum creatinine: 12.14 mg/dL), extreme elevations in muscle enzymes (creatine kinase: 250,000 IU/L), and cardiac biomarkers (troponin-T: 543 ng/L). Initial management did not improve his condition. On day 15, thyroid function tests confirmed non-autoimmune hypothyroidism (TSH: 55.58 μIU/mL, free T4: 0.06 ng/dL).

**Interventions:**

The patient underwent hemodialysis and conservative treatment initially, followed by levothyroxine replacement therapy on day 16.

**Outcomes:**

Renal function, urine output, and muscle enzyme levels gradually improved after thyroid hormone supplementation. By discharge on day 40, the patient’s serum creatinine decreased to 3.08 mg/dL, and hemodialysis was discontinued. At 7 months posttreatment, his renal function stabilized at CKD stage G3bA2 with normal thyroid function.

**Lessons:**

This case highlights the importance of considering hypothyroidism in patients with unexplained rhabdomyolysis and persistent cardiac symptoms, particularly in those with CKD. Early thyroid function testing can lead to timely treatment and improved outcomes in such complex cases.

## 1. Introduction

Rhabdomyolysis is characterized by skeletal muscle cell damage resulting in the release of toxic intracellular material into the bloodstream. Major causes include trauma, ischemia, drugs, toxins, metabolic disorders, and infections.^[1]^ Hypothyroidism commonly presents with symptoms such as myalgia, muscle stiffness, easy fatigability, and occasional mild myopathy with elevated muscle enzymes. Rarely, hypothyroidism may lead to rhabdomyolysis, characterized by the rapid destruction of skeletal muscle and release of myoglobin, creatine kinase (CK), urate, and electrolytes into the circulation.^[2]^ Thyroid diseases can also affect the heart’s structure and function, with diastolic dysfunction being a common abnormality in hypothyroidism. Although uncommon, hypothyroidism-induced cardiomyopathy and systolic dysfunction may occur, particularly as an initial manifestation of hypothyroidism.^[3]^ Cases of thyroid disease-associated myocarditis are rare and most commonly reported in autoimmune thyroiditis, such as Hashimoto’s disease and autoimmune polyglandular syndrome.^[4–6]^

This is the first reported case of acute kidney injury due to non-autoimmune hypothyroidism-induced rhabdomyolysis and myocarditis.

## 2. Case presentation

### 2.1. Chief complaints

A 71-year-old male was hospitalized due to myalgia and anorexia that developed over a few days after overworking on the farm.

### 2.2. History of present illness

The patient complained of intermittent left chest pain for 1 month as well as dull epigastric pain for a few days accompanied by myalgia, anorexia, and decreased urine volume for 2 days. Anuria developed 1 day prior to presentation.

### 2.3. History of past illness

The patient has been taking metformin for diabetes mellitus (DM) and candesartan 60 mg/d for hypertension for 8 years. In addition, he has been on aspirin 100 mg/d and rosuvastatin 10 mg/d following right coronary artery stenting due to non-ST elevated myocardial infarction 2 months after being diagnosed with DM and hypertension. Furthermore, he has been receiving nilotinib 300 mg/d for 5 years for chronic myelogenous leukemia, achieving a confirmed complete molecular response for 2 years. Three years prior to presentation, he was diagnosed with chronic kidney disease (CKD) due to DM nephropathy. At the most recent assessment, his CKD stage was G3aA3, with a serum creatinine level of 1.47 mg/dL, an estimated glomerular filtration rate using the CKD-EPI equation of 48 mL/min/1.73 m², and a urine protein-to-creatinine ratio of 0.5 mg/mg (Table 1).

**Table 1:** Initial laboratory findings.

	Variable	Element	Before 40 days	Initial day	Reference
Serum	WBC	×10^3^/mm^3^	6.82	12.16	4.0–10.0
	Hemoglobin	g/dL	9.4	10.1	13.0–17.0
	Hematocrit	%	33	29	39–52
	Platelet	×10^3^/mm^3^	303	275	130–400
	BUN	mg/dL	18.0	112.0	6–20
	Creatinine	mg/dL	1.47	12.14	0.6–1.2
	eGFR (CKD-EPI)	mL/min/1.73 m^2^	48	4	
	Calcium	mg/dL	9.6	7.9	8.6–10.2
	Phosphorus	mg/dL	3.0	8.8	2.7–4.5
	Glucose	mg/dL	97	92	70–110
	Uric acid	mg/dL	4.1	11	3.4–7.0
	Sodium	mmol/L	139.4	133.6	135–145
	Potassium	mmol/L	4.8	7.8	3.3–5.1
	Chloride	mmol/L	106.0	102.7	98–110
	Total CO_2_	mmol/L	25	10	21–30
	AST	IU/L	40	733	0–37
	ALT	IU/L	26	419	0–41
	CK	IU/L	ND	250,000	0–190
	LDH	IU/L	243	1879	135–225
	CRP	mg/L	0.1	1.7	0–5
	CK-MB	ng/mL	ND	289.0	0–4.9
	NT-proBNP	pg/mL	ND	3758	0–125
	Troponin-T	ng/L	ND	543	<14
	Osmolality	mosm/kg	334		276–300
Urine	S.G.	–	1.017	1.018	1.005–1.030
	Protein	–	1+	2+	–
	RBC	/HPF	>100	<1	0–4
	WBC	/HPF	<1	1–4	0–4
	Pr/Cr ratio	–	0.5	0.2	–
	Sodium	mmol/L	ND	49.8	–
	Potassium	mmol/L	ND	35.7	–
	Chloride	mmol/L	ND	27.1	–
	Osmolality	mosm/kg	ND	343	300–900

ALT = alanine aminotransferase, AST = aspartate aminotransferase, BUN = blood urea nitrogen, CK = creatine kinase, CRP = C-reactive protein, eGFR = estimated glomerular filtration rate, LDH = lactate dehydrogenase, ND = not done, RBC = red blood cell, S.G. = specific gravity, WBC = white blood cell.

### 2.4. Physical examination

Upon assessment, the patient’s blood pressure was 137/78 mm Hg, heart rate 151 beats/min, and body temperature 36.4 °C. Clear lung sounds were noted bilaterally, and pitting edema was not observed. In terms of muscle strength, his upper extremities demonstrated full strength (5/5) and lower extremities exhibited slightly diminished strength (4/4) upon arrival at the emergency room.

### 2.5. Initial laboratory findings

The initial laboratory results revealed a sudden worsening of azotemia with serum creatinine level elevated to 10.19 mg/dL. In addition, elevated muscular enzyme levels were observed. The patient also presented with hyponatremia (serum Na^+^ 133.6 mEq/L), hyperkalemia (serum K^+^ 7.8 mEq/L), and high anion gap metabolic acidosis (serum Cl^−^ 102.7 mEq/L, total CO_2_ 10 mEq/L). Notably, serum lactic acid level was within the normal range at 1.70 mmol/L (reference range: 0.5–2.2 mmol/L) (Table 1).

### 2.6. Further diagnostic work-up and treatment

**Figure 1.**
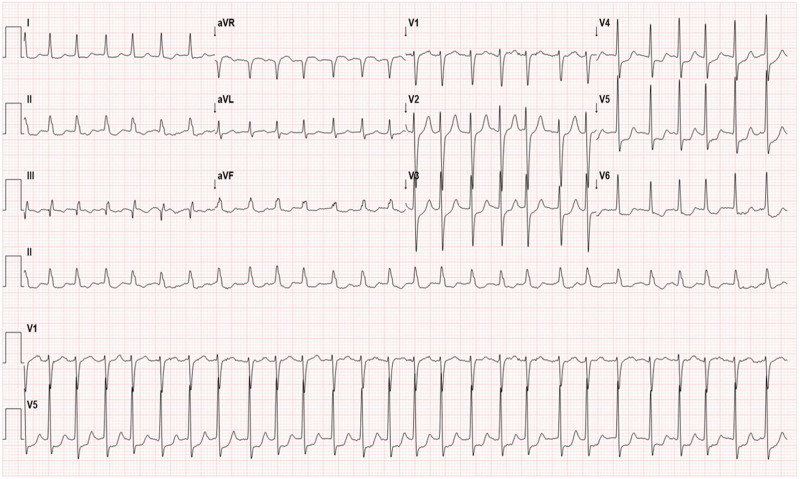
Initial electrocardiogram showing atrial fibrillation with rapid ventricular response and ST-T wave abnormality.

Reoccurrence of non-ST elevated myocardial infarction was suspected due to changes in the electrocardiogram, transitioning from first-degree AV block to atrial fibrillation with rapid ventricular response (Fig. 1), accompanied by elevated cardiac enzymes (troponin-T level of 543 ng/L; reference, <14 ng/L). A transthoracic echocardiogram revealed persistent ischemic insult in the right coronary artery territory. However, coronary angiography showed an intact stent and no significant changes compared with coronary angiography 8 years prior.

**Figure 2.**
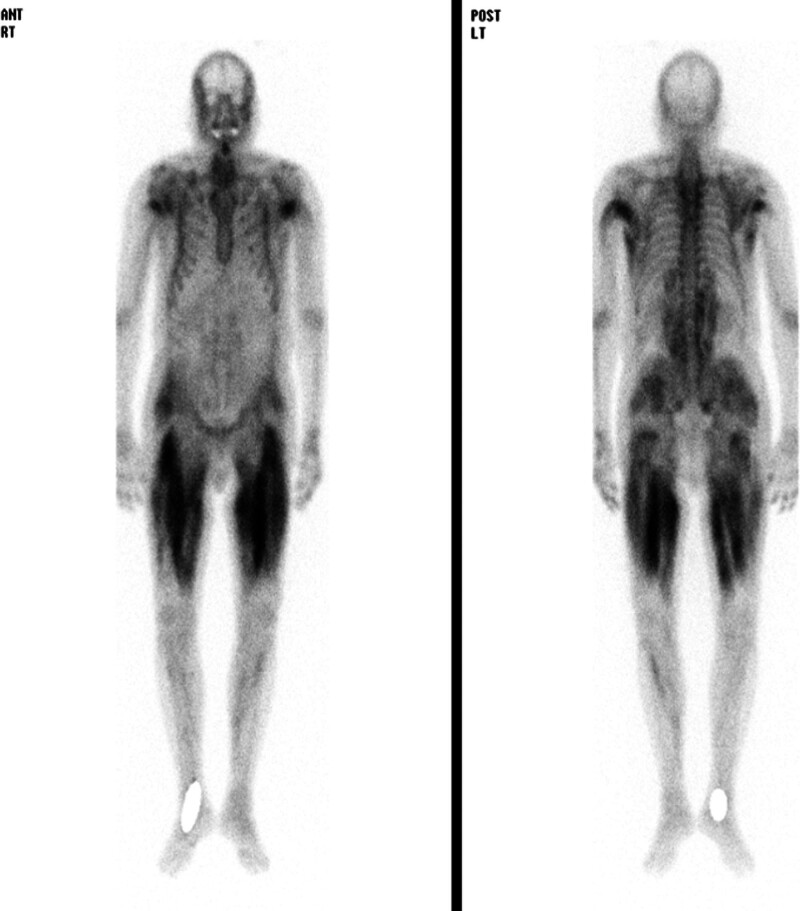
Bone scan revealing increased tracer uptake in both thighs and buttocks and the upper arm muscles.

A bone scan showed heterogeneously increased tracer uptake in various areas, including the thighs, buttocks, shoulders, axilla, mid-back paravertebral region, masseter, and temporalis (Fig. 2).

Due to oliguria and uremic gastropathy, the patient underwent emergent hemodialysis. Acute kidney injury secondary to vigorous exercise-induced rhabdomyolysis was suspected.

Despite receiving hemodialysis 3 times per week for 2 weeks and maintaining absolute bed rest, the patient’s muscular pain persisted, with elevated CK and lactate dehydrogenase and no improvement in oliguria and azotemia. Autoimmune myopathy was considered a possible underlying cause, prompting the initiation of steroid therapy (1 mg/kg) for 1 week following the biopsy of the right rectus femoris muscle. However, neither muscular nor cardiac enzyme levels improved, and the muscle biopsy did not indicate inflammation.

Thyroid function tests conducted on day 15 after admission revealed overt hypothyroidism (free T4: 0.06 ng/dL, reference, 0.93–1.70 ng/dL; TSH: 55.58 μIU/mL, reference, 0.27–4.2 μIU/mL; total T3: 20.33 mg/dL, reference, 80–200 mg/dL), and thyroglobulin Ab, anti-TPO Ab, and TSH-R Ab levels were normal. So, the patient was diagnosed with non-autoimmune hypothyroidism. Levothyroxine (LT4) 75 μg was initiated on admission day 16. Serum creatinine level decreased to 6.8 mg/dL, and urine output increased to 400 mL/d by admission day 20. Rehabilitation treatment was maintained, and steroid dosage was reduced.

**Figure 3.**
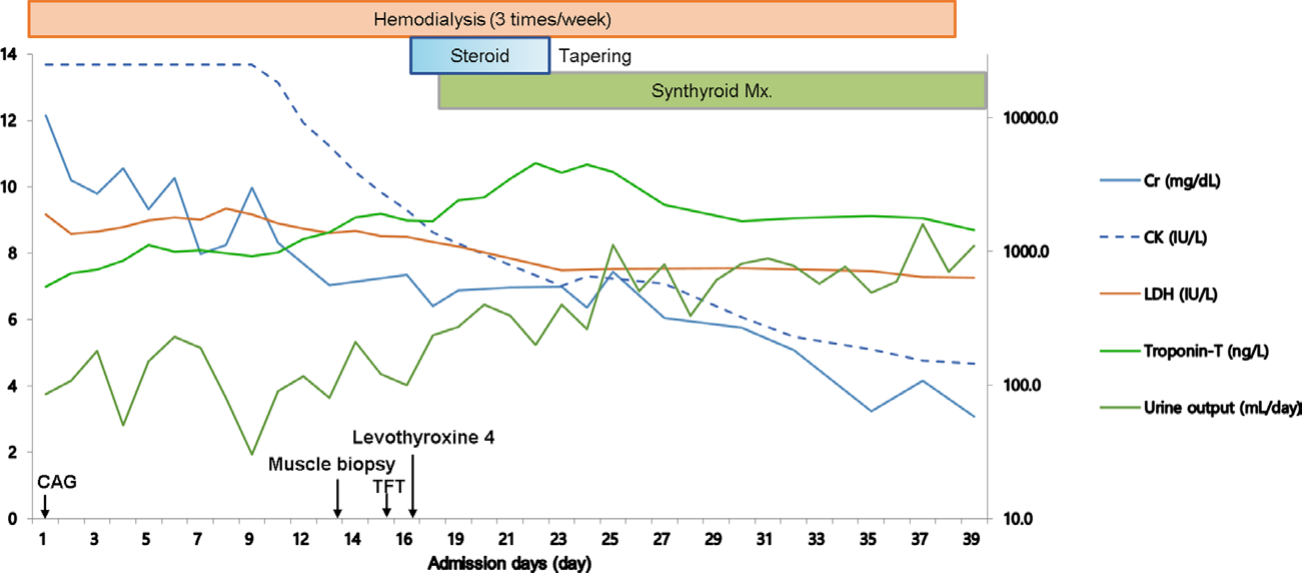
Clinical course. CAG = coronary angiography, Cr = serum creatinine, CK = creatine kinase, LDH = lactate dehydrogenase, TFT = thyroid function test.

On admission day 39, hemodialysis was discontinued as urine output had increased to 1100 mL/d and serum creatinine level decreased to 3.08 mg/dL. The patient was discharged on admission day 40 (Fig. 3).

### 2.7. Outcome and follow-up

After 4 weeks of thyroxine replacement therapy, the patient’s chest pain and muscular symptoms resolved, and thyroid function tests normalized. Seven months post-discharge, his serum creatinine stabilized at 1.92 mg/dL, with normalization of muscular (CK: 152 IU/L, lactate dehydrogenase: 296 IU/L) and cardiac enzyme levels (troponin-T: 376 ng/L). A follow-up electrocardiogram showed recovery to normal sinus rhythm. Eight months after onset, the patient remains on LT4 therapy with stable renal function at CKD stage G3bA2, a urine protein-to-creatinine ratio of 0.3 mg/mg, serum creatinine of 1.91 mg/dL, and an estimated glomerular filtration rate of 34 mL/min/1.73 m², alongside maintained normal thyroid function.

## 3. Discussion

We presented a rare case of acute kidney injury (AKI) induced by rhabdomyolysis and myocarditis due to non-autoimmune hypothyroidism in a patient with CKD. The occurrence of AKI in CKD patients can accelerate CKD progression and increase mortality rates, making accurate identification of the underlying causes and proper management crucial.^[7]^ CKD patients are at an increased risk for electrolyte imbalances, such as hyperphosphatemia and hypocalcemia, which may make them more susceptible to developing rhabdomyolysis. CKD has also been identified as an independent risk factor for rhabdomyolysis.^[8]^ Several studies have epidemiologically indicated an increased risk of hypothyroidism in patients with CKD.^[9,10]^ Case reports on rhabdomyolysis caused by hypothyroidism have been rarely documented, yet clear clinical evidence linking hypothyroidism and rhabdomyolysis specifically in CKD patients remains limited.^[11,12]^ However, several studies have epidemiologically indicated an increased risk of hypothyroidism in patients with CKD.^[9,10]^

Hypothyroidism can contribute to the development of rhabdomyolysis through various mechanisms, including changes in muscle fiber composition, impaired mitochondrial activity, and dysregulation of metabolic pathways such as the Krebs cycle and fatty acid catabolism.^[13]^ Although the exact cause of rhabdomyolysis in hypothyroidism remains unclear, impaired mitochondrial oxidative metabolism and glycogenolysis may play a role.^[14,15]^

Common symptoms of hypothyroidism include fatigue, weight gain, sensitivity to colds, constipation, and cognitive decline, which are often observed in elderly CKD patients. As a result, the diagnosis of hypothyroidism may be delayed. However, suppose a CKD patient presents with muscle weakness along with the symptoms mentioned above and severely elevated serum levels of muscle enzymes. In that case, performing thyroid function tests early to check for thyroid hormone deficiency is important. This can lead to rapid alleviation of symptoms and improvement in renal prognosis through timely thyroid hormone administration.^[16,17]^ Our case patient experienced a lack of improvement in muscle enzyme levels despite more than 2 weeks of conservative treatment, leading to a deterioration of kidney function that required dialysis. However, after thyroid hormone supplementation, we observed a gradual recovery of both muscle enzyme levels and kidney function.

Another severe complication in our patient was myocarditis. Thyroid hormones significantly influence the cardiovascular system, affecting every structure of the heart and its specialized conducting system. Dysfunction of the thyroid system has been associated with various cardiovascular conditions, including atrial fibrillation, atherosclerosis, hypertension, dyslipidemia, sinus bradycardia and tachycardia, atrioventricular block, torsades de pointes, pericarditis, pericardial effusion, left ventricular systolic and diastolic dysfunction, cardiomyopathy, and mitral valve prolapse.^[18]^

Diagnosis of myocarditis is typically confirmed based on cardiac muscle biopsy and MRI.^[19]^ Significant associations between hypothyroidism and endomyocardial biopsy-proven chronic autoimmune myocarditis have been reported.^[20]^ In clinical practice, myocarditis can be diagnosed based on clinical improvement in myocardial enzyme levels following normalization of thyroid function. Myocarditis has also been reported in patients with low T3 syndrome, with Hashimoto’s thyroiditis being a common underlying thyroid disease suspected to contribute to the pathophysiology through chronic inflammation affecting the heart, muscles, and thyroid.^[20,21]^ In our patient, all thyroid-related autoantibodies were within normal ranges, leading to the diagnosis of non-autoimmune hypothyroidism. This suggests that inflammation affecting multiple organs can occur even in non-autoimmune hypothyroidism.

Improvement of rhabdomyolysis associated with hypothyroidism is generally reported to occur within 6 months to 1 year after LT4 treatment,^[15]^ and our patient took 7 months to recover.

## 4. Conclusion

If there is slow recovery from rhabdomyolysis despite adequate conservative treatment, hypothyroidism should be considered as part of the differential diagnosis. Our case has demonstrated that untreated hypothyroidism can lead to serious life-threatening complications such as AKI and myocarditis. In particular, in elderly CKD patients with multiple underlying conditions, it may be difficult to suspect thyroid disease based solely on symptoms. Therefore, we recommend early thyroid function tests as a screening tool in cases of unexplained rhabdomyolysis. We hope that our case serves as evidence that highlights the importance of identifying and managing thyroid disease, not only in CKD patients but also in the diagnosis and management of patients with rhabdomyolysis.

## Acknowledgments

The authors extend their sincere gratitude to the patient involved in this case and appreciate the dedication of the entire medical team contributing to this study.

## Author contributions

**Conceptualization:** Hyejin Jeon, Hyun-Jung Kim.

**Data curation:** Hyejin Jeon, Seunghye Lee, Sehyun Jung.

**Supervision:** Hyun-Jung Kim.

**Validation:** Seunghye Lee, Sehyun Jung, Hani Jang, Se-Ho Chang.

**Writing – original draft:** Hyejin Jeon, Seunghye Lee.

**Writing – review & editing:** Hyun-Jung Kim.
